# Experimental Investigation of Optimal Relay Position for Magneto-Inductive Wireless Sensor Networks

**DOI:** 10.3390/s20092720

**Published:** 2020-05-10

**Authors:** Gang Qiao, Muhammad Muzzammil, Niaz Ahmed, Irfan Ullah

**Affiliations:** 1 Acoustic Science and Technology Laboratory, Harbin Engineering University, Harbin 150001, China; qiaogang@hrbeu.edu.cn (G.Q.); muzzammilm@hrbeu.edu.cn (M.M.); 2 Key Laboratory of Marine Information Acquisition and Security (Harbin Engineering University), Ministry of Industry and Information Technology, Harbin 150001, China; 3 College of Underwater Acoustic Engineering, Harbin Engineering University, Harbin 150001, China; 4 College of Information and Communication Engineering, Harbin Engineering University, Harbin 150001, China; khanirfan44sw@hrbeu.edu.cn

**Keywords:** magneto-Induction (MI), resonance, waveguide, optimal relay position, magneto-inductive wireless sensor networks (MIWSNs)

## Abstract

Magneto-inductive (MI) waveguide technology is often proposed to increase the MI communication distance without adding significant cost and power consumption to the wireless sensor network. The idea is to add intermediate relaying nodes between transmitter (Tx) and receiver (Rx) to relay the information from Tx to Rx. Our study of MI wave-guides has realized that adding a relay node improves the communication distance, however, the performance is greatly dependent on the position of the relaying node in the network. We therefore, in this work have investigated the effect of placement of a relay node and have determined the optimal relay position. We have performed various sets of experiments to thoroughly understand the behavior and identified three main regions: (a) for region 1, when the distance between Tx and Rx is equal or less than the diameter of the coils (d≤2r), the optimal relay position is close to Tx, (b) for region 2, when the distance between Tx and Rx is greater than diameter of the coils but less than twice the diameter (2r<d<4r), the optimal relay position lies in the center of Tx and Rx, and (c) for region 3, when the distance between the Tx and Rx is equal or greater than twice the diameter of the coils (d≥4r), the optimal relay position is close to Rx.

## 1. Introduction

Magneto-induction (MI) communication has recently emerged as an alternative choice for underground and underwater wireless sensor network applications [1,2,3], because of easy penetration, instant speed, high data rates, stable and predictable channel response. Furthermore, MI communication offers a unique advantage of inter-medium communication such as air-to-water/soil and water/soil-to-air due to the same magnetic permeability of air, and water [4,5]. Due to its unique advantages [1,3], MI communication unfolds a wide range of applications and attract researchers to study different areas of MI communications such as coil designing [6,7,8], directionality [1,6,7,8], transmission distance [4,5,9,10], localization [11,12,13], routing [5,14,15] and MAC protocols [1,16].

In a magneto-inductive wireless sensor network (MIWSN), any two nodes (Tx and Rx) communicate with each other by means of magnetic field. Tx creates a magnetic field and Rx couples to the magnetic fields. The communication distance between Tx and Rx depends on strength of the magnetic field (MFS). MFS is proportional to the magnetic moment *m*, which is defined as $m=\mu_{0}\mu_{r}NIA$, where $\mu_{o}=4\pi \times 10^{-7}$ H/m is the magnetic permeability constant, $\mu_{r}$ is relative permeability of the medium, *N* is number of turns, I(t) is the current flowing through the Tx coil, and *A* is area of the Tx coil. Magnetic field decays rapidly with the sixth power of the distance ($\frac{1}{d^{6}}$), and thus limits the communication distance.

**Figure 1:**
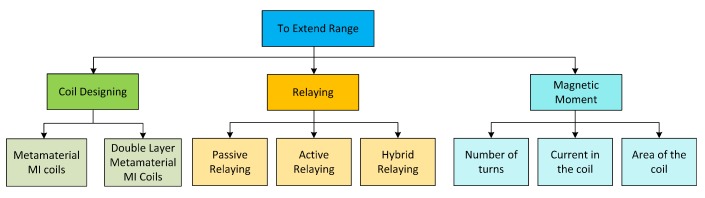
Various techniques that can be applied to extend the communication distance.

To increase the communication distance, Tx needs to create a stronger magnetic moment by increasing one or more than one of the three parameters (*N*, *I* and *A*). However, all of these parameters can not be increased to an indefinite extent, for example, increasing the current will add more power consumption. Similarly, increasing area of the coil will end up in huge size structures which may not be feasible for some applications. A stronger magnetic field can also be created by modifying some physical parameters of the coils as proposed by Guo et.al (use of metamaterial magnetic induction) and Sharma et.al (use of double layer metamaterial magnetic induction) [7,17]. However, this is achieved on the expense of cost and complex designs. On the other hand, a cheaper and easy solution proposed in literature is the relaying technique [4,5,9,18]. Relaying can be active, passive (waveguide) or hybrid and has proven to be a viable option to extend communication distance. Active relaying add more power consumption to the overall network but hybrid and waveguide techniques improve the communication distance with minimal power consumption. The different methods used in the literature to increase the communication range are summarized in Figure 1.

MI waveguide technique uses intermediate relay nodes and has been investigated in various dimensions in the literature. In microwave bandpass filters [19,20,21,22], waveguide resonators are used to achieve narrow bandwidth with better insertion losses and high power handling capabilities [22]. Similarly, in wireless power transfer (WPT), the waveguide technique (relay resonators or repeaters) is used to transfer power over longer distances and achieve maximum transfer efficiency [23,24,25,26]. This paper, however, focuses the study of MI waveguide technique to communicate over longer distance without adding additional computational processing or power consumption to the network. The idea is that nodes already present in the sensor network can act as relay nodes and help to relay the information from one point (Tx) to the other (Rx).

MI waveguide based communication has been investigated and considered useful to increase the communication distance, however our study shows that this may not always be the case, as placement of relay coil plays significant role in the performance. Placing relay coil at some places may boost up the performance while at other places may degrade the performance. This is therefore really important to investigate the effect of placement of relay coils. We thus perform extensive sets of experiments to study the relaying behavior and figure out the regions where the performance is optimal. Knowing the optimal region will help in better utilization of the resources in a wireless sensor network and extend the range of applications such as mine disaster rescue, intelligent agriculture, locating AUVs, underwater structural health monitoring, oil and gas pipeline monitoring and so forth [3,9,27]. Furthermore, our study has identified that there are three different regions and are dependent on the diameters of the Tx coils. For region 1, when the distance between Tx and Rx is equal or less than the diameter of the coils (d≤2r), the optimal relay position is close to Tx. For region 2, when the distance between Tx and Rx is greater than diameter of the coils but less than twice the diameter (2r<d<4r), the optimal relay position lies in the center of Tx and Rx. For region 3, when the distance between the Tx and Rx is equal or greater than twice the diameter of the coils (d≥4r), the optimal relay position is close to Rx.

The rest of the paper is organized as follows. In Section 2, the related work is presented. In Section 3, MI waveguide based communication system model, resonance frequency and optimal relay position are discussed. Experimental setup, results and analysis are presented in Section 4. Finally, in Section 5, the paper is concluded.

## 2. Related Work

As discussed in Section 1, MI waveguide technique offers help to increase the communication distance and has been presented in literature with different aspects. In References [4,5] the MI waveguide technique are studied for underground and underwater environment respectively. In Reference [5], the author presented the channel modeling for MI waveguide based communication in underwater environment and compared the performance in fresh and sea water. From the simulation results, the author concluded that, with the help of MI waveguide technique the communication distance could greatly be improved in fresh water compared with the seawater. In their study, the distance between the MI transmitter, receiver and the relay nodes were assumed to be equally spaced from each other. In References [4,28], Sun et. al. This work also showed the significance of the MI waveguide technique in terms of achieving more communication distance, however the distance between the transmitter and receiver was short and the relay nodes were again equally spaced from each other. Similarly, in Reference [27] a testbed of MI waveguide based communication has been presented in underground environment where the authors deployed six equally spaced relay nodes between the MI transmitter and receiver. The received signal strength was improved by 10dBm with six relay nodes for a communication distance of 2m. If the optimal regions were known and applied, the similar improvement in received signal strength could be achieved with a smaller number of relay nodes. In Reference [9], Sun et. al presented MI waveguide based relay coils deployment strategy for one-dimensional network along the underground pipelines. They determined the optimal number of relay nodes for each link based on the required bandwidth and transmission link length. Similarly, the optimal network throughput of MI based communication with and without relays has been studied by the identifying optimal system parameters, topology and deployment strategy in Reference [10]. The authors concluded that the MI waveguide based communication performed poor when the average communication distance between the nodes (transmitter, receiver or relay) was not too large. However, the study lacked to provide the information about the average communication distance between the nodes.

From the literature review of MI waveguide based communication, we have identified that most of the existing works has been done in simulations and there lacks practical evaluation of the performance. Furthermore, all the studies have assumed to place the relaying coils equidistant and that too in the middle. Intuitively, this looks a fairly reasonable assumption but interestingly, the position of placing the relaying coil is not that simple and require thorough investigation. Placing the relaying coil at the center may not always improve the performance, rather decouples the mutual coupling and lower the performance. Therefore, in this work, we investigate the optimal position for the relay coils by performing laboratory experiments and give a better insight and understanding to the research community.

## 3. MI Waveguide Technique

In this section, we first present and explain the principle of MI waveguide technique. We next explain the importance of resonance frequency and the effect of relaying coils on resonance frequency. At the end of the section, we present the theoretical model and present the three different regions for optimal placement of the relay coil.

### 3.1. System Model

**Figure 2:**
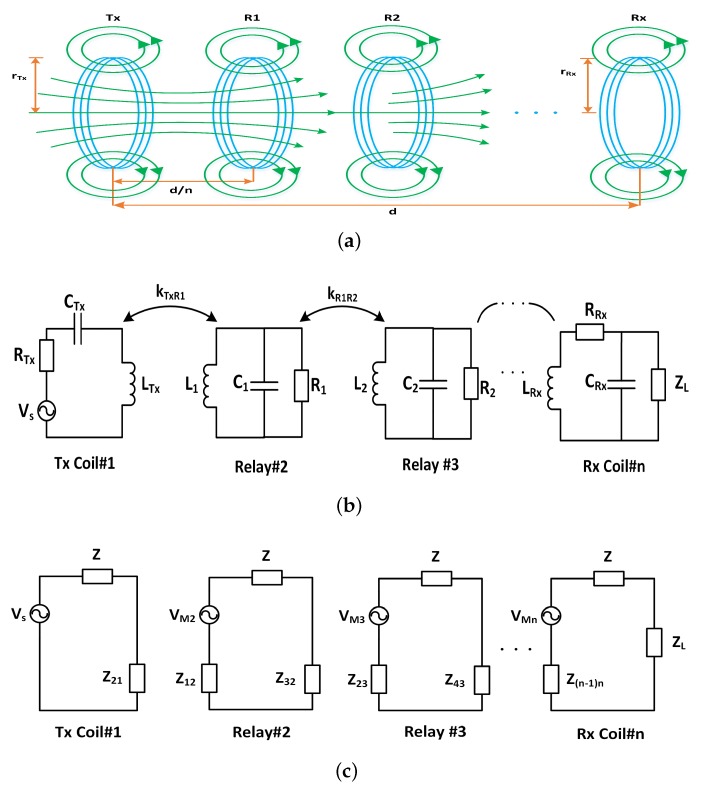
Magneto-inductive (MI) waveguide based communication system. (**a**) MI waveguide model, (**b**) multistage transformer model, and (**c**) equivalent circuit model.

Figure 2a shows a concept diagram of MI waveguides where relay coils are co-axially placed between Tx and Rx coils [4,5]. $r_{Tx}$ and $r_{Rx}$ are the radius of Tx and Rx coils respectively, $\frac{d}{n}$ is the distance between any two coils (Tx, Rx or relay coils), and *d* is the total transmission distance of the MI waveguide based communication system. The basic idea of waveguide technique is based on the MI principle where a time varying electric signal in Tx coil generates a varying magnetic field around the Tx coil. This varying magnetic field couples with a neighbouring coil and induces voltage in the neighbouring coil. Since the magnetic field created around the Tx coil may not exist for longer distances, the idea is to add intermediate relaying coils between the Tx and Rx coil. So the Tx coil will relay the information to R1, and R1 will relay the information to R2 and the phenomena will go on until the information is relayed to Rx. This way by adding more and more relaying nodes in the network, the communication distance can be increased farther and farther.

The MI waveguide coils can be modeled as multistage transformer model, as shown in Figure 2b, where any two nodes (Tx, Rx, or relay coils) can be modeled as the primary and secondary coils of a transformer and are extensively used in the study of microwave bandpass filters and WPT [4,29,30]. $V_{s}$ denotes the voltage supplied to the transmitter, $C_{Tx},C_{R1},C_{R2},\cdots ,C_{Rx}$ are the capacitors in each coil. The link between two coupled coils is denoted by coupling coefficient, which is $k=\frac{M_{ij}}{\sqrt{LiLj}}$, where $M_{ij}$, (i≠j) is the mutual inductance of any two coupled coils, Li, and $L_{j}$ denote the self-inductance of any two coils *i* and *j* respectively [31]. The equivalent circuit of multistage transformer model is shown in Figure 2c, where the received power $P_{Rx}$ can be written as [4]

(1) $$\begin{array}{c} P_{Rx}=ℜ\left\{\frac{Z_{L}\times V_{Mn}^{2}}{{\left(Z_{(n-1)n}+Z+Z_{L}\right)}^{2}}\right\}, \end{array}$$

where $Z=R+j\omega L+\frac{1}{j\omega C}$, ω is the angular frequency, *L* is the inductance of coil, *C* is capacitance of a capacitor, while $V_{Mn}$ is the induced voltage at the receiver coil, and $Z_{L}$ denotes the load impedance of the receiver.

When the coils are resonant, the capacitor is designed to satisfy the condition of $j\omega L+\frac{1}{j\omega C}=0$ and the induced voltage in this case can be written as [4]

(2) $$\begin{array}{c} V_{Mn}=V_{s}\times \frac{-j\omega M}{R}\times \frac{-j\omega M}{R+\frac{\omega^{2}M^{2}}{R}}\times \frac{-j\omega M}{R+\frac{\omega^{2}M^{2}}{R+\frac{\omega^{2}M^{2}}{R}}}\cdots \frac{-j\omega M}{R+\frac{\omega^{2}M^{2}}{R+\frac{\omega^{2}M^{2}}{R+\cdots }}}, \end{array}$$

where *R* is the resistance of the coil and *M* is the mutual inductance given by [9] as

(3) $$\begin{array}{c} M≃\frac{\mu \pi N_{Tx}N_{R1}r_{Tx}^{2}r_{R1}^{2}}{2{\left(\frac{d}{n}\right)}^{3}}. \end{array}$$

The path loss of MI waveguide based communication depends on the communication distance *d*, required bandwidth *B* and number of coils (Tx, Rx and relays) *n*, which can be written as [4,9]

(4) $$\begin{array}{c} PL_{MI-relay}\left(d,n,\omega \right)≃6.02+20log\zeta \left(\frac{Z}{\omega M},n\right). \end{array}$$

### 3.2. Resonance Frequency of the System

As shown in Figure 2b the MI multistage transformer model uses a LC circuit that acts as a resonator. The resonance phenomena coupled with the induction make MI communication more promising and results in longer range. The resonance frequency for the MI coupled coils as provided in the WPT literature [32] is given as

(5) $$\begin{array}{c} \omega =\frac{1}{\sqrt{C_{Tx}L_{Tx}}}=\frac{1}{\sqrt{C_{R1}L_{R1}}}=\cdot \cdot \cdot =\frac{1}{\sqrt{C_{Rx}L_{Rx}}}, \end{array}$$

where ω is the angular resonance frequency, $C_{Tx}$ and $L_{Tx}$ are the capacitance and inductance of the transmitter node, $C_{R_{1}}$ and $L_{R_{1}}$ are the capacitance and inductance of the first relay node, and $C_{Rx}$ and $L_{Rx}$ are the capacitance and inductance of the receiver node respectively. For relaying coils it has been studied previously in terms of maximum power transfer [23,32,33], that the resonant frequency for all the coils in the network splits into a set of different resonant frequencies, depending on odd/even numbers of intermediate relay nodes [23]. For even number of relays, the resonance frequency is different than the original resonant frequency between Tx and Rx, however for odd number of relays, the original resonant frequency still exists. It therefore is really important to avoid using even number of relaying nodes in a network as the resonance frequency will shift and the communication will be significantly affected. We therefore, in this work consider odd number of relays only, and the set of resonant frequencies can then be calculated by applying Kirchhoff voltage law (KVL) as shown in Figure 2c, and can be written as [23]

$$\left(1-\frac{\omega_{o}^{2}}{\omega_{r}^{2}}\right)I_{Tx}+k_{TxR1}I_{R1}\sqrt{\frac{L_{R1}}{L_{Tx}}}=0$$

$$k_{TxR1}I_{Tx}\sqrt{\frac{L_{Tx}}{L_{R1}}}+\left(1-\frac{\omega_{o}^{2}}{\omega_{r}^{2}}\right)I_{R1}+k_{R1R2}I_{R2}\sqrt{\frac{L_{R2}}{L_{R1}}}=0$$

(6) $$k_{R1R2}I_{R1}\sqrt{\frac{L_{R1}}{L_{R2}}}+\left(1-\frac{\omega_{o}^{2}}{\omega_{r}^{2}}\right)I_{R2}+k_{R2R3}I_{R3}\sqrt{\frac{L_{R3}}{L_{R2}}}=0$$

⋮

$$k_{(n-1)n}I_{R(n-1)}\sqrt{\frac{L_{R(n-1)}}{L_{Rx}}}+\left(1-\frac{\omega_{o}^{2}}{\omega_{r}^{2}}\right)I_{Rx}=0.$$

In Equation (7), the coupling coefficient between the adjacent coil will be strong as compared with the non-adjacent coils (i.e., $k_{TxR1}≃k_{R1R2}\gg k_{TxR2}$), therefore, they are assumed negligible. Re-arranging Equation (7), we get

(7) $$\begin{array}{c} \frac{\omega_{o}^{2}}{\omega_{r}^{2}}\left[\begin{array}{c} I_{Tx}\\ I_{R1}\\ I_{R2}\\ \vdots \\ I_{Rx} \end{array}\right]=\left[\begin{array}{ccccc} 1 & k_{TxR1}I_{R1}\sqrt{\frac{L_{R1}}{L_{Tx}}} & 0 & \cdots & 0\\ k_{TxR1}I_{Tx}\sqrt{\frac{L_{Tx}}{L_{R}1}} & 1 & k_{R1R2}I_{R2}\sqrt{\frac{L_{R2}}{L_{R1}}} & \cdot & 0\\ 0 & k_{R1R2}I_{R1}\sqrt{\frac{L_{R1}}{L_{R}2}} & 1 & \cdots & 0\\ \vdots & \vdots & \vdots & \cdots & \vdots \\ 0 & 0 & 0 & k_{(n-1)n}I_{R(n-1)}\sqrt{\frac{L_{R(n-1)}}{L_{Rx}}} & 1 \end{array}\right]\left[\begin{array}{c} I_{Tx}\\ I_{R1}\\ I_{R2}\\ \vdots \\ I_{Rx} \end{array}\right], \end{array}$$

where

(8) $$\begin{array}{c} \frac{\omega_{o}^{2}}{\omega_{r}^{2}}=1+\sqrt{k_{TxR1}}+k_{R1R2},1,1+\sqrt{k_{R(n-2)R(n-1)}+k_{R(n-1)Rx}}, \end{array}$$

are the eigenvalues and here *n* is the total number of odd relay nodes in the MI waveguide based communication system, and the new resonant frequencies $\omega_{r}$ can be calculated from (8), which can be written as [23]

(9) $$\begin{array}{c} \omega_{r}=\frac{\omega_{o}}{\sqrt{1+\sqrt{k_{TxR1}+k_{R1R2}}}},\omega_{o},\frac{\omega_{o}}{\sqrt{1-\sqrt{k_{R(n-2)R(n-1)}}+k_{R(n-1)Rx}}}. \end{array}$$

### 3.3. Optimal Relay Position

To extend the communication distance between Tx and Rx, a number of relay coils can be inserted between them. However, it is important to know where the relay coils can be placed. As previously, the MI waveguide is being used and proposed, however the relays have always been placed either in the center or at equidistance. Our laboratory experiments on the other hand showed that placing the relaying coil in the center can act as a repeater and help to strengthen the magnetic field, but that may not be the optimal position. In fact, there are some distance points where the magnetic filed strength can be increased considerably. The factors contribute to find the optimal position for the relay coils depend on the radius of the Tx coil, and the total distance between the Tx and Rx. The resonant frequency is also a key factor, and it can be noted that only an odd number of relay coils can be inserted between Tx and Rx. We have identified that there exists three different regions and are explained below.

#### 3.3.1. Region 1

**Figure 3:**
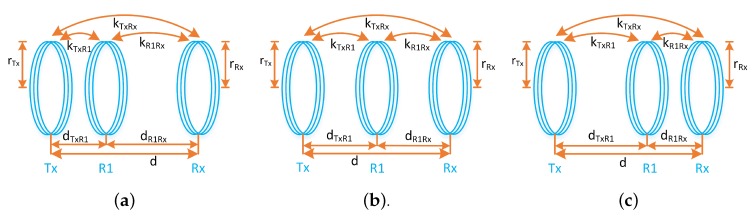
Three different regions for optimal relay position, (**a**) region 1, (**b**) region 2, and (**c**) region 3.

Let *d* be the communication distance between the transmitter and receiver, $r_{Tx}=r_{Rx}=r_{R1}$ be the radius of Tx, Rx and relay coil. It has been found that when *d* is less or equal to the diameter of the coil radius, that is, $2r_{Tx}$, then the optimal relay position exist near transmitter as shown in Figure 3a. The distance between the transmitter and relay is represented by $d_{TxR1}$. The mutual inductance *M* between Tx and relay coil will be significant in this case and can be written as [34]:

(10) $$\begin{array}{c} M\left(d\leq 2r\right)≃\frac{\mu \pi N_{Tx}N_{R1}r_{Tx}^{2}r_{R1}^{2}}{2d_{TxR1}^{3}}. \end{array}$$

Here, we assume that $N_{Tx}=N_{R1}=N_{Rx}=N$, and $r_{Tx}=r_{R1}=r_{Rx}=r$, then (10) becomes

(11) $$\begin{array}{c} M\left(d\leq 2r\right)≃\frac{\mu \pi N^{2}r^{4}}{2d_{TxR1}^{3}}. \end{array}$$

#### 3.3.2. Region 2

When the communication distance between the transmitter and receiver is greater than diameter of the coils but less than twice the diameter of the coils, that is, 2r<d<4r, the optimal relay position exist in the middle of transmitter and receiver as shown in Figure 3b. In this region, the mutual inductance *M* will be same in adjacent coils, which can be written as

(12) $$\begin{array}{c} M\left(2r<d<4r\right)≃\frac{\mu \pi N^{2}r^{4}}{2d_{TxR1}^{3}}. \end{array}$$

Here $d_{TxR1}=d_{R1Rx}$ and optimal relay position is simply $\frac{d}{2}$. Hence (12) becomes

(13) $$\begin{array}{c} M\left(2r<d<4r\right)≃\frac{\mu \pi N^{2}r^{4}}{2{\left(\frac{d}{2}\right)}^{3}}. \end{array}$$

#### 3.3.3. Region 3

When the communication distance is equal to or greater than twice the diameter of the coil, that is, d≥4r, the optimal relay position exist near the receiver as shown in Figure 3c. The mutual inductance for this region can be written as

(14) $$\begin{array}{c} M\left(d\geq 4r\right)≃\frac{\mu \pi N^{2}r^{4}}{2d_{R1Rx}^{3}}. \end{array}$$

## 4. Experimental Evaluation

In this section, we first present the laboratory experimental setup and then provide analysis of the results in detail.

### 4.1. Experimental Setup

**Figure 4:**
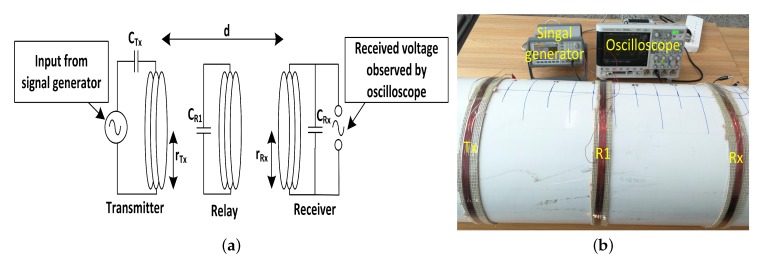
Experimental setup for MI waveguide based communication system, (**a**) schematic representation, and (**b**) lab setup.

Laboratory experiments were performed to show the relaying behavior in the three different regions. Figure 4 shows the experimental setup—a schematic representation of the setup is shown in Figure 4a and lab setup is shown in Figure 4b. A function generator was used to provide time varying current to the transmit coil (Tx), and an oscilloscope was used to record the received voltage at the receive coil (Rx). A relay coil (R1) was also placed between the Tx and Rx which was moved from Tx to Rx to observe the response at Rx. All the coils were wounded around a plastic pipe to maintain a perfect symmetry and alignment between the Tx/Rx/R1 coils. Different set of experiments were performed by changing the number of turns and radius of the coils to show the consistent relaying behavior in the three different regions. Pipes with radius of *r* = 6 cm, *r* = 8 cm, *r* = 10 cm and *r* = 15 cm were used to perform the relaying experiments. We performed each experiment with different number of turns: N=30,20and10. All the coils were tuned to a fixed resonant frequency for each setup (Table 1) by adding capacitors ($C_{Tx}=C_{R1}=C_{Rx}$ = 5.6 nF) to each coil.

### 4.2. Results Analysis

**Figure 5:**
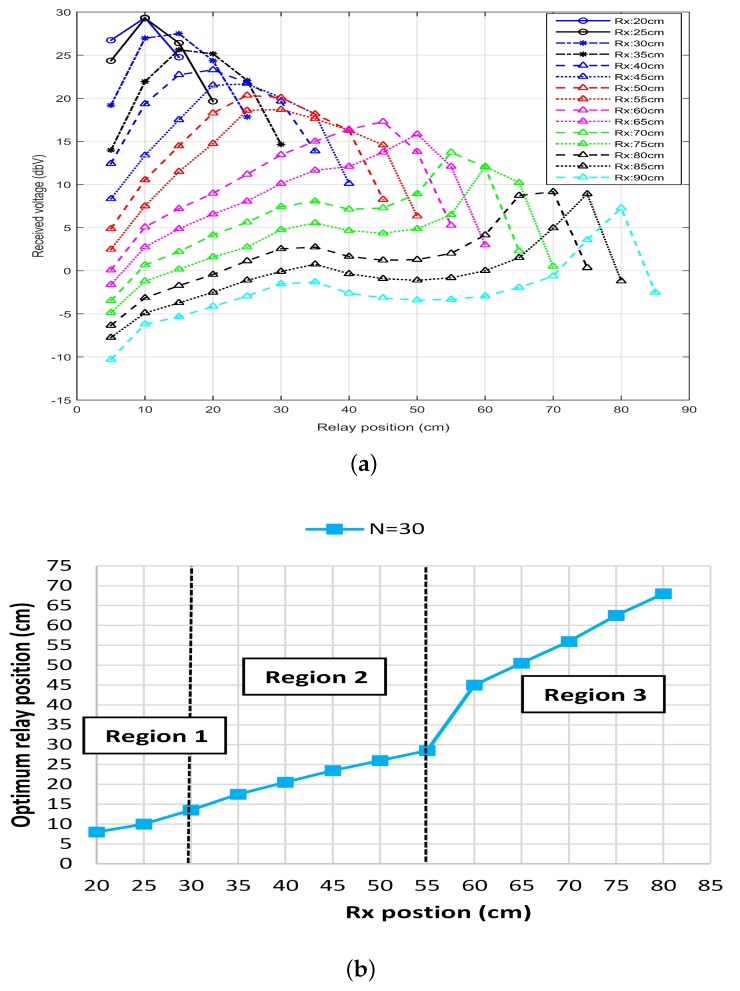
Received voltage and optimal relay position when *r* = 15 cm and *N* = 30, (**a**) received voltage vs. relay position under different receiver positions, and (**b**) optimal relay position vs. receiver positions.

Figure 5a shows the results for the experiment performed with coil of radius *r* = 15 cm and number of turns as $N_{Tx}=N_{R1}=N_{Rx}=30$. The Tx was placed at 0 cm and Rx was initially placed at 20 cm. The relay was then inserted between the Tx and Rx. R1 at first was placed close to the Tx and then moved towards Rx. The voltage at the Rx was recorded and the effect of the relaying coil can be seen in the Figure 5a. The Rx was then moved to 25 cm and the whole process of moving the R1 from Tx to Rx was repeated. After each set of experiment, Rx was moved away with a regular interval of 5 cm and the whole process was repeated.

It can clearly be seen from the results, that there exists a distance point where each curve has a peak voltage and we call this distance point as the optimal relay position. From the different curves shown in Figure 5a, it can be seen that when the distance between Tx and Rx is equal or less than diameter of the Tx coil (until Rx = 30 cm), the optimal relay position is close to the Tx. Similarly, if the distance between Tx and Rx is less than twice the diameter of the Tx coil (until Rx = 55 cm), the optimal relay position is exactly at the center. Furthermore, if the distance between Tx and Rx exceeds twice the diameter of Tx coil (Rx > 60 cm), then the optimal relay position is closed to the Rx coil instead of the center. For more clear representation, the data is plotted in the Figure 5b where x-axis represent the position of the Rx while the y-axis represent the optimal position of R1. The three different regions are clearly marked and can easily be identified from the results.

**Figure 6:**
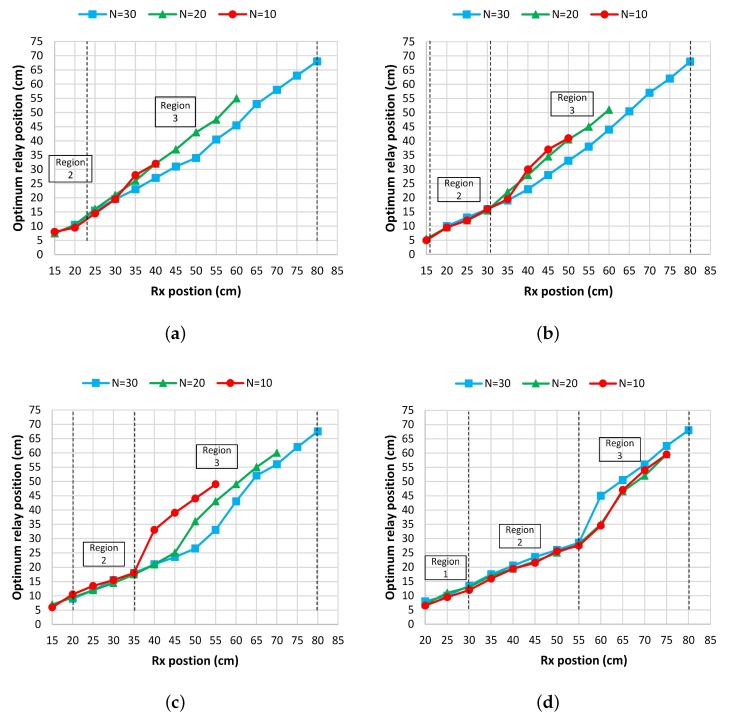
Optimal relay position vs. receiver position under different number of turns when: (**a**) r=6 cm, (**b**) r=8 cm, (**c**) r=10 cm, and (**d**) r=15 cm.

To validate the presence of three different regions with the relaying nodes, we repeated the set of experiments with coils of different radii and different number of turns. The setup for all the different set of experiments was the same where we fixed Tx at 0 cm and then placed Rx at 15 cm (in some cases 20 cm). R1 was then placed between Tx and Rx, and the optimal relay position for R1 was figured out. Figure 6a–d shows the optimal relay positions of R1 with respect to Rx position for radius of *r* = 6 cm, *r* = 8 cm, *r* = 10 cm, and *r* = 15 cm respectively. It can clearly be seen that when R1 is placed between Tx and Rx, the optimal position for the R1 is not always in the center, rather there exists three different regions. For region 1, the optimal relay position exists near the transmitter. For region 2, the optimal relay position exists in the middle. For region 3, the optimal relay position exists near the receiver. It can further be noted that the regions are proportional to the diameter of the Tx coil. The region 1 exists for the distance equal or less than the diameter of the Tx coil. The region 2 lies in the area when the distance is greater than the diameter of the Tx coil and less than twice the diameter of the coil. Similarly region 3 starts for distance equal or greater than twice the diameter of the Tx coil.

**Figure 7:**
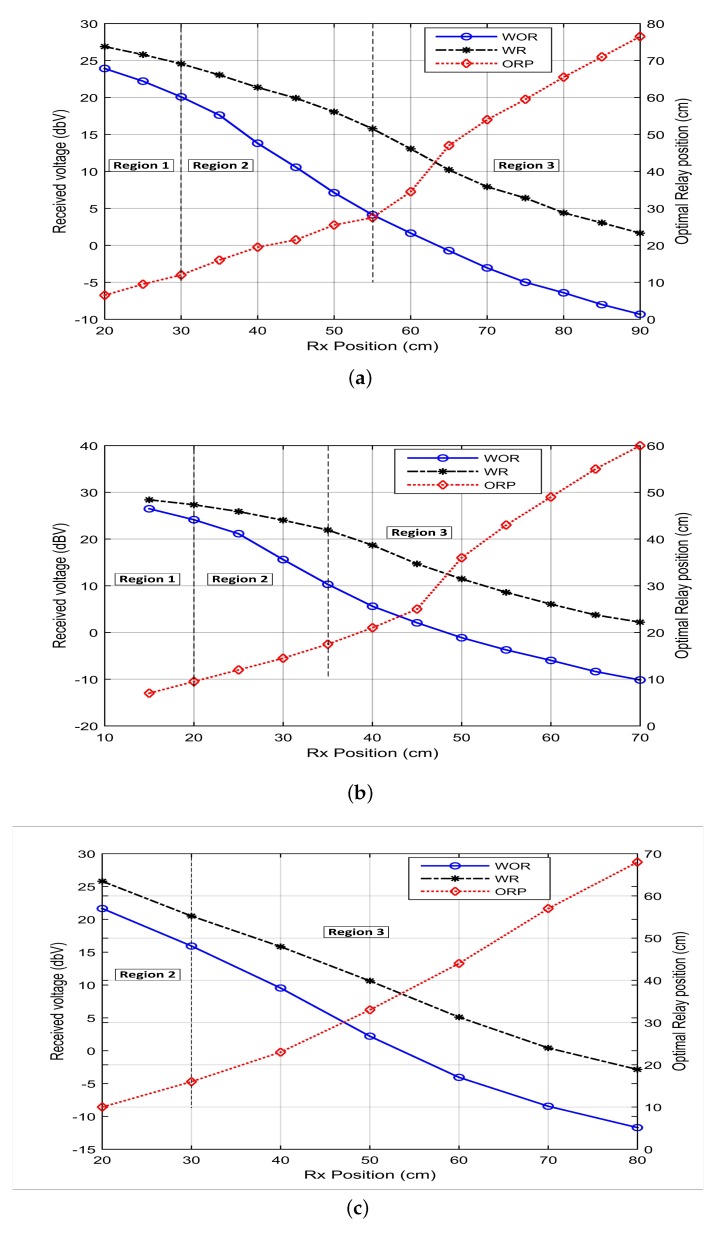
Received voltage with and without relay vs. receiver position when: (**a**) r=15 cm and N=10, (**b**) r=10 cm and N=20, and (**c**) r=8 cm and N=30.

In Figure 7, the received voltage without relay (WOR), with relay (WR), and the optimal relay position with respect to various Rx positions are shown. In Figure 7a–c the received voltage WOR and WR is shown with respect to Rx position when: N=10 and $r_{Tx}=r_{R1}=r_{rx}$ = 15 cm, N=20 and $r_{Tx}=r_{R1}=r_{rx}$ = 10 cm, and N=30 and $r_{Tx}=r_{R1}=r_{rx}=8$ cm respectively. Further, the optimal relay position with respect to Rx position is shown too. It can be analyzed from Figure 7a,b, when the communication distance is smaller or equal to coil diameter, that is, region 1, the received voltage without relay is quite high and there may not be need of relay nodes. However, when the communication distance increases from the coil diameter, that is, region 2 and especially region 3, the received voltage decreases rapidly. From Figure 7a, it can be observed that for region 3, when Rx position is 90 cm, the received voltage without relay is approx −10 dBV, while it is enhanced by more than 10 dBV with the MI waveguide based communication system. Furthermore, from Figure 7b, it can be analyzed that for region 3, when Rx position 70 cm, the received voltage without relay is -10 dBV, while it is enhanced by more then 10 dBV with the MI waveguide based communication system. Similarly, the received voltage at receiver position of 80 cm is enhanced by 10 dBV with MI waveguide based communication as compared to MI communication without relaying in case of N=30 and r= 8 cm as shown in Figure 7c. Therefore, from the analysis of Figure 7, it is suggested that for region 2 and region 3, one should use the MI waveguide based communication system.

## 5. Conclusions

In this paper, an optimal relay position for an MI waveguide based communication system is investigated. Three different regions are identified—for d≤2r, the optimal relay position exist near the transmitter. For 2r<d<4r, the optimal relay position exist in the middle of the transmitter and receiver, while for d≥4r, the optimal position of relay shift to the receiver. Laboratory experiments with different number of turns and area of the coils are carried out to validate the identified regions. Furthermore, the performance comparison of MI communication with no relay and with a single relay under optimal relay position is presented. It is suggested that, for identified region 2 and 3, the MI waveguide based communication is useful in terms of achieving more communication distance.

## Figures and Tables

**Table 1:** Resonance frequencies for four different set of configurations.

Sets	Resonance Frequency		
	*N* = 30	*N* = 20	*N* = 10
*r* = 6 cm	160 KHz	219 KHz	443 KHz
*r* = 8 cm	126 KHz	174 KHz	325 KHz
*r* = 10 cm	109 KHz	147 KHz	273 KHz
*r* = 15 cm	81 KHz	119 KHz	216 KHz

## References

[1] Akyildiz I.F., Wang P., Sun Z. (2015). Realizing underwater communication through magnetic induction. IEEE Commun. Mag..

[2] Kisseleff S., Akyildiz I.F., Gerstacker W.H. (2018). Survey on advances in magnetic induction-based wireless underground sensor networks. IEEE Internet Things J..

[3] Li Y., Wang S., Jin C., Zhang Y., Jiang T. (2019). A Survey of underwater magnetic induction communications: Fundamental issues, recent advances, and challenges. IEEE Commun. Surv. Tutor..

[4] Sun Z., Akyildiz I.F. (2010). Magnetic induction communications for wireless underground sensor networks. IEEE Trans. Antennas Propag..

[5] Domingo M.C. (2012). Magnetic induction for underwater wireless communication networks. IEEE Trans. Antennas Propag..

[6] Ahmed N., Radchenko A., Pommerenke D., Zheng Y.R. (2018). Design and evaluation of low-cost and energy-efficient magneto-inductive sensor nodes for wireless sensor networks. IEEE Syst. J..

[7] Guo H., Sun Z. M^2^I communication: From theoretical modeling to practical design. Proceedings of the 2016 IEEE International Conference on Communications (ICC).

[8] Muzzammil M., Babar Z., Niaz A., Qiao G., Liu S. Directivity Pattern of Different Coil Structures for Magneto-Coupled Communication Systems. Proceedings of the OCEANS 2019 MTS/IEEE Marseille.

[9] Sun Z., Wang P., Vuran M.C., Al-Rodhaan M.A., Al-Dhelaan A.M., Akyildiz I.F. (2011). MISE-PIPE: Magnetic induction-based wireless sensor networks for underground pipeline monitoring. Ad Hoc Netw..

[10] Kisseleff S., Akyildiz I.F., Gerstacker W.H. (2014). Throughput of the magnetic induction based wireless underground sensor networks: Key optimization techniques. IEEE Trans. Commun..

[11] Schmidt H.K., Akyildiz I.F., Lin S.C., Al-Shehri A.A. (2017). Magnetic Induction based Localization for Wireless Sensor Networks in Underground Oil Reservoirs. U.S. Patent Application.

[12] Huang H., Zheng Y.R. (2018). 3-D localization of wireless sensor nodes using near-field magnetic-induction communications. Phys. Commun..

[13] Tan X., Sun Z., Wang P., Sun Y. (2020). Environment-aware localization for wireless sensor networks using magnetic induction. Ad Hoc Netw..

[14] Menon K.U., Vikas V., Hariharan B. Wireless power transfer to underground sensors using resonant magnetic induction. Proceedings of the 2013 Tenth International Conference on Wireless and Optical Communications Networks (WOCN).

[15] Sun Z., Akyildiz I.F. (2013). Optimal deployment for magnetic induction-based wireless networks in challenged environments. IEEE Trans. Wirel. Commun..

[16] Ahmed N., Hoyt J., Radchenko A., Pommerenke D., Zheng Y.R. A Multi-Coil Magneto-Inductive Transceiver for Low-Cost Wireless Sensor Networks. Proceedings of the Underwater Communications Networking Conference.

[17] Sharma P., Meena R.S., Bhatia D. Analytical channel model for double layer metamaterial-improved magnetic induction communication. Proceedings of the 2017 International Conference on Advances in Computing, Communications and Informatics (ICACCI).

[18] Masihpour M., Franklin D., Abolhasan M. (2013). Multihop relay techniques for communication range extension in near-field magnetic induction communication systems. J. Netw..

[19] Hunter I., Rhodes J.D. (1982). Electronically tunable microwave bandpass filters. IEEE Trans. Microw. Theory Tech..

[20] Tyurnev V.V. (2010). Coupling coefficients of resonators in microwave filter theory. Prog. Electromagn. Res..

[21] Musonda E., Hunter I.C. (2015). Microwave bandpass filters using re-entrant resonators. IEEE Trans. Microw. Theory Tech..

[22] Arnold C., Parlebas J., Zwick T. (2014). Reconfigurable waveguide filter with variable bandwidth and center frequency. IEEE Trans. Microw. Theory Tech..

[23] Ahn D., Hong S. (2012). A study on magnetic field repeater in wireless power transfer. IEEE Trans. Ind. Electron..

[24] Saha C., Anya I., Alexandru C., Jinks R. (2018). Wireless power transfer using relay resonators. Appl. Phys. Lett..

[25] Guo K., Zhou J., Sun H., Yao P. Design Considerations for a Position-Adaptive Contactless Underwater Power Deliver System. Proceedings of the 2019 22nd International Conference on Electrical Machines and Systems (ICEMS).

[26] Hasaba R., Okamoto K., Yagi T., Kawata S., Eguchi K., Koyanagi Y. High Efficient Wireless Power Transfer System for AUV with Multiple Coils and Ferrite under Sea. Proceedings of the 2019 IEEE Wireless Power Transfer Conference (WPTC).

[27] Tan X., Sun Z., Akyildiz I.F. (2015). Wireless underground sensor networks: MI-based communication systems for underground applications. IEEE Antennas Propag. Mag..

[28] Sun Z., Akyildiz I.F. Underground wireless communication using magnetic induction. Proceedings of the 2009 IEEE International Conference on Communications.

[29] Leszczynska N., Szydlowski L., Mrozowski M. (2013). A novel synthesis technique for microwave bandpass filters with frequency-dependent couplings. Prog. Electromagn. Res..

[30] Kung M.L., Lin K.H. (2017). Dual-band coil module with repeaters for diverse wireless power transfer applications. IEEE Trans. Microw. Theory Tech..

[31] Wang C.S., Stielau O.H., Covic G.A. (2005). Design considerations for a contactless electric vehicle battery charger. IEEE Trans. Ind. Electron..

[32] Sample A.P., Meyer D.T., Smith J.R. (2011). Analysis, experimental results, and range adaptation of magnetically coupled resonators for wireless power transfer. IEEE Trans. Ind. Electron..

[33] Hong J.S., Lancaster M.J. (1996). Couplings of microstrip square open-loop resonators for cross-coupled planar microwave filters. IEEE Trans. Microw. Theory Tech..

[34] Frankl D.R. (1986). Electromagnetic Theory.

